# Supplementary material for: Macroscopic Synovial Inflammation Correlates with Symptoms and Cartilage Lesions in Patients Undergoing Arthroscopic Partial Meniscectomy: A Clinical Study
Source: J Clin Med. 2022 Jul 26;11(15):4330. doi: 10.3390/jcm11154330 (PMC9330366; doi:10.3390/jcm11154330)
Supplement: Supplementary file 1 [file jcm-11-04330-s001.zip › jcm-1728196-supplementary.pdf]

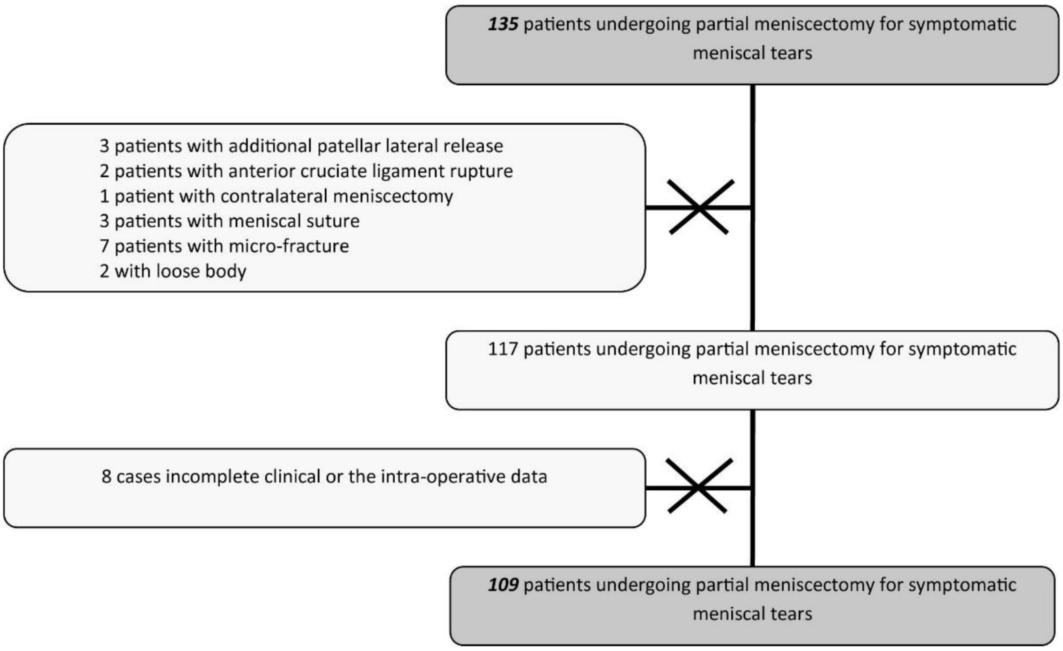

Figure S1: Flowchart of patients enrolled in the study.

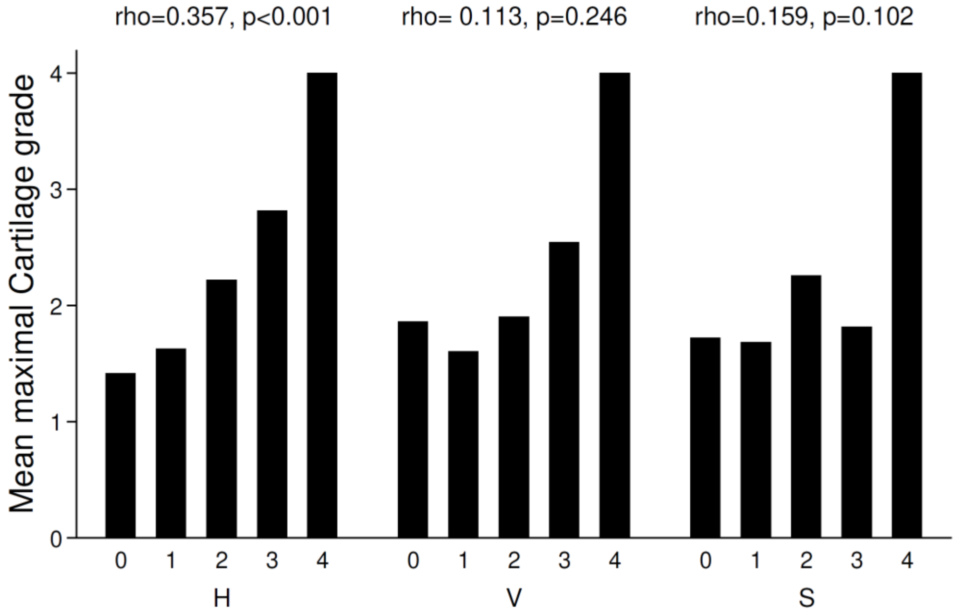

Figure S2. Correlation between severity of chondral damage and suprapatellar synovial inflammation component (H = hyperplasia, V = vascularity, S = synovitis). Correlation was evaluated with Spearman rho.

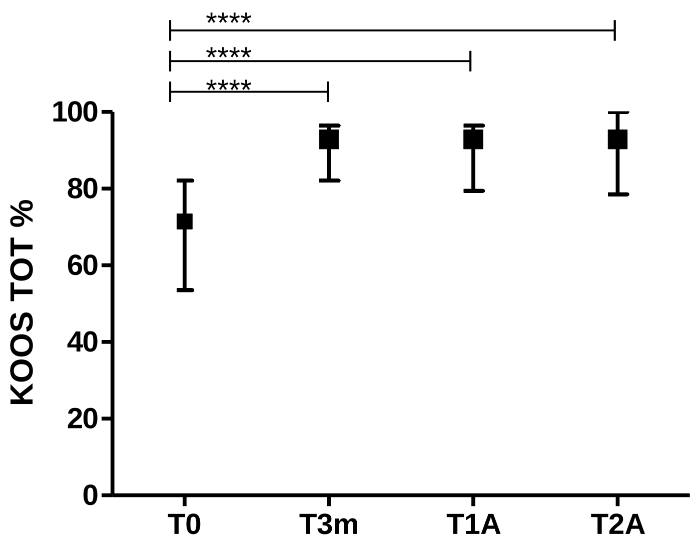

Figure S3. Improvement in total KOOS at three months (T3m) post-operatively, which was maintained at one (T1A) and two years (T2A) of follow-up. KOOS TOT = Total Knee injury and Osteoarthritis Outcome Score. **** $p < 0.0001$

Table S1. **Synovial inflammation characteristics (0-4) and subtotal synovial score at the medial/lateral and peri-ACL compartents (0-12) (N = 109)**

**Synovial inflammation characteristics of medial gutter, number (%)**					
	0	1	2	3	4
Hypertrophy	41 (38)	38 (35)	22 (20)	6 (5)	2 (2)
Vascularity	44 (40)	30 (28)	27 (25)	7 (6)	1 (1)
Synovitis	43 (39)	30 (28)	29 (27)	6 (5)	1 (1)
**Subtotal synovial score, number (%)**					
	Absent *0*	Mild *(≤ 3)*	Mild-moderate *(4-6)*	Moderate *(7-9)*	Severe *(≥10)*
	30 (28)	34 (31)	39 (35)	5 (5)	1 (1)
**Synovial inflammation characteristics of lateral gutter, number (%)**					
	0	1	2	3	4
Hypertrophy	45 (41)	41 (38)	19 (17)	3 (3)	1 (1)
Vascularity	47 (43)	39 (36)	17 (16)	6 (5)	0 (0)
Synovitis	43 (39)	30 (27)	29 (27)	6 (6)	1 (1)
**Subtotal synovial score, number (%)**					
	Absent *0*	Mild *(≤ 3)*	Mild-moderate *(4-6)*	Moderate *(7-9)*	Severe *(≥10)*
	29 (27)	48 (44)	28 (26)	4 (4)	0 (0)
**Synovial inflammation characteristics of peri-ACL, number (%)**					
	0	1	2	3	4
Hypertrophy	43 (39)	39 (36)	21 (19)	5 (5)	1 (1)
Vascularity	40 (37)	33 (30)	26 (24)	10 (9)	0 (0)
Synovitis	50 (46)	30 (27)	24 (22)	4 (4)	1 (1)
**Subtotal synovial score, number (%)**					
	Absent *0*	Mild *(≤ 3)*	Mild-moderate *(4-6)*	Moderate *(7-9)*	Severe *(≥10)*
	26 (24)	48 (44)	30 (27)	5 (5)	0 (0)

Table S2. Patients Characteristics by etiology of meniscal lesion: degenerative versus traumatic (N= 109)

Factor	Level	Total	Degenerative	Trauma	p-value
**Patients number**		109	62	47	
**Age, median (IQR)**		49 (40, 56)	51.5 (44, 57)	43 (33, 54)	**0.002**
**MALE (1)**	0	35 (32.1%)	23 (37%)	12 (26%)	0.20
	1	74 (67.9%)	39 (63%)	35 (74%)	
**BMI, median (IQR)**		27 (23.8, 29.8)	27.4 (23.8, 30.3)	26.9 (22.9, 29.4)	0.16
**Right knee (1)**	0	53 (48.6%)	28 (45%)	25 (53%)	0.41
	1	56 (51.4%)	34 (55%)	22 (47%)	
**Symtomps duration, median (IQR) years**		0.77 (0.32, 1.52)	1 (0.5, 2)	0.41 (0.25, 0.91)	***<0.001**
**MED1LAT2BIL3**	1	79 (72.5%)	44 (71%)	35 (74%)	***0.005**
	2	14 (12.8%)	4 (6%)	10 (21%)	
	3	16 (14.7%)	14 (23%)	2 (4%)	
**CART DEG GRADE_TOT**	0	26 (24.3%)	12 (20%)	14 (30%)	*0.074*
	1	10 (9.3%)	3 (5%)	7 (15%)	
	2	33 (30.8%)	20 (33%)	13 (28%)	
	3	25 (23.4%)	15 (25%)	10 (22%)	
	4	13 (12.1%)	11 (18%)	2 (4%)	
**Meniscal lesion extension**	0-1 cm	22 (20.2%)	17 (27%)	5 (11%)	*0.065*
	1-2 cm	56 (51.4%)	27 (44%)	29 (62%)	
	>2 cm	31 (28.4%)	18 (29%)	13 (28%)	
**SYN_YES (1)**	0	7 (6.4%)	3 (5%)	4 (9%)	0.44
	1	102 (93.6%)	59 (95%)	43 (91%)	
**SUPRA H**	≤2	96 (88.1%)	53 (85%)	43 (91%)	0.34
	≥3	13 (11.9%)	9 (15%)	4 (9%)	
**SUPRA V**	≤2	97 (89.0%)	55 (89%)	42 (89%)	0.91
	≥3	12 (11.0%)	7 (11%)	5 (11%)	
**SUPRA S**	≤2	96 (88.1%)	55 (89%)	41 (87%)	0.81
	≥3	13 (11.9%)	7 (11%)	6 (13%)	
**SUPRA TOT**	≤2	34 (31.2%)	19 (31%)	15 (32%)	0.62
	3-4	35 (32.1%)	18 (29%)	17 (36%)	
	≥5	40 (36.7%)	25 (40%)	15 (32%)	
**lc_h**	≤2	105 (96.3%)	59 (95%)	46 (98%)	0.46
	≥3	4 (3.7%)	3 (5%)	1 (2%)	
**lc_v**	≤2	103 (94.5%)	59 (95%)	44 (94%)	0.73
	≥3	6 (5.5%)	3 (5%)	3 (6%)	
**lc_s**	≤2	103 (94.5%)	59 (95%)	44 (94%)	0.73
	≥3	6 (5.5%)	3 (5%)	3 (6%)	
**lc_tot**	≤2	53 (48.6%)	30 (48%)	23 (49%)	***0.036**
	3-4	36 (33.0%)	16 (26%)	20 (43%)	
	≥5	20 (18.3%)	16 (26%)	4 (9%)	
**mc_h**	≤2	101 (92.7%)	55 (89%)	46 (98%)	0.069
	≥3	8 (7.3%)	7 (11%)	1 (2%)	
**mc_v**	≤2	101 (92.7%)	56 (90%)	45 (96%)	0.28
	≥3	8 (7.3%)	6 (10%)	2 (4%)	
**mc_s**	≤2	102 (93.6%)	57 (92%)	45 (96%)	0.42
	≥3	7 (6.4%)	5 (8%)	2 (4%)	
**mc_tot**	≤2	47 (43.1%)	25 (40%)	22 (47%)	0.79
	3-4	29 (26.6%)	17 (27%)	12 (26%)	
	≥5	33 (30.3%)	20 (32%)	13 (28%)	
**peri_h**	≤2	103 (94.5%)	57 (92%)	46 (98%)	0.18
	≥3	6 (5.5%)	5 (8%)	1 (2%)	

**peri_v**	≤2	99 (90.8%)	55 (89%)	44 (94%)	0.38
	≥3	10 (9.2%)	7 (11%)	3 (6%)	
**peri_s**	≤2	104 (95.4%)	58 (94%)	46 (98%)	0.29
	≥3	5 (4.6%)	4 (6%)	1 (2%)	
**peri_tot**	≤2	51 (46.8%)	31 (50%)	20 (43%)	0.72
	3-4	29 (26.6%)	15 (24%)	14 (30%)	
	≥5	29 (26.6%)	16 (26%)	13 (28%)	
**BL_KOOSSYM, median (IQR)**		71.43 (53.57, 82.14)	71.43 (57.14, 78.57)	67.86 (50, 82.14)	0.89
**BL_KOOSPAIN, median (IQR)**		63.89 (50, 75)	61.11 (50, 75)	63.89 (50, 75)	0.58
**BL_KOOSADL, median (IQR)**		69.12 (51.47, 85.29)	66.915 (51.47, 83.82)	72.06 (51.47, 85.29)	0.81
**BL_KOOSSP, median (IQR)**		35 (10, 50)	30 (10, 55)	35 (5, 50)	0.95
**BL_KOOSQOL, median (IQR)**		43.75 (25, 56.25)	43.75 (25, 56.25)	37.5 (31.25, 56.25)	0.94

BMI = body max index; MED = medial; LAT = lateral; BIL = bilateral 3; CART DEG = cartilage degeneration; cm = centimeters; SYN = synovitis; SUPRA H = suprapatellar hyperplasia, SUPRA V = suprapatellar vascularity; SUPRA S = suprapatellar synovitis; **SUPRA TOT** = suprapatellar subtotal score; lc_h = lateral compartment hyperplasia; lc_v = lateral compartment vascularity; lc_s = lateral compartment synovitis; lc_tot = lateral compartment subtotal score; mc_h = medial compartment hyperplasia; mc_v = medial compartment vascularity; mc_s = medial compartment synovitis; **mc_tot** = medial compartment subtotal score; peri_h = peri-anterior cruciate ligament hyperplasia; peri_v = anterior peri-cruciate ligament vascularity; peri_s = peri-anterior cruciate ligament synovitis; **peri_tot** = peri-anterior cruciate ligament subtotal score; BL = basal; KOOS = Knee injury and Osteoarthritis Outcome Score; ADL = activity in daily living; SSP = function in sport and recreation; QOL = Quality of life; IQR = interquartile range; *p<0.05
